# Bacterial extracellular vesicle as a predictive biomarker for postoperative delirium status after spinal surgery: a prospective cohort study

**DOI:** 10.1097/JS9.0000000000003024

**Published:** 2025-08-27

**Authors:** Jai J. Jee, Sujung Park, Jeongmin Kim, Hyangkyu Lee, Hong Koh, Bon-Nyeo Koo

**Affiliations:** a Department of Pediatrics, Yonsei University College of Medicine, Severance Fecal Microbiota Transplantation Center, Severance Hospital, Seoul, Republic of Korea; b Department of Anesthesiology and Pain Medicine, Asan Medical Center, University of Ulsan College of Medicine, Seoul, Republic of Korea; c Department of Anesthesiology and Pain Medicine, Anesthesia and Pain Research Institute, Yonsei University College of Medicine, Seoul, Republic of Korea; d Mo-Im Kim Nursing Research Institute, College of Nursing, Yonsei University, Seoul, Republic of Korea

**Keywords:** biomarker, delirium, extracellular vesicle, prognosis, random forest, surgery

## Abstract

**Background:**

Prognostic factors significantly associated with postoperative delirium (POD) have been reported discordantly, possibly due to heterogeneous cohorts. Here, bacteria extracellular vesicles (BEVs) were introduced to predict the POD status of a unique patient cohort.

**Methods:**

One hundred twenty-eight patients who underwent spinal surgery participated in this prospective cohort study. Significant preoperative factors (i.e., baseline characteristics, and sequences of 16s rRNA genes from bloods and stools) between patients with and without delirium were subjected to random forest classifiers for prediction model, and potential metabolites that regulate the POD were inferred *in silico*.

**Results:**

No significant differences were found between patients with and without delirium in terms of demographics, anthropometrics, intervention history or preoperative cognitive function scores, except for circulating BEVs; delirium group had less diverse BEVs dominated with EVs from Gammaproteobacteria, whereas more diverse BEVs enriched with EVs from Bacilli and Alphaproteobacteria were significantly associated with non-delirium. Compared to that with baseline characteristics or gut microbiome, prediction model using random forest classifier with the significant BEVs yielded the lowest error rate of 21.59%, and was validated with an independent data set, resulting in 80% accuracy. Moreover, EVs from Moraxellaceae and Acinetobacter showed the highest probabilities of prediction of the POD despite their low relative abundance, indicating the most significant prognostic markers for the POD. As the inference of a potential metabolites that regulate the POD, succinate and enterobacterial common antigens delivered from BEV cargo were expected to participate in pathogenic events, whereas S-methyl-5ʹ-thioadenosine, 2-oxoglutarate, pyruvate, acetate and butyrate may play a neuroprotective role in the POD.

**Conclusions:**

The profile of circulating preoperative BEVs is the key prognostic factor for distinguishing POD in elderly surgical patients with controlled baseline conditions. Metabolites of defensive and offensive mechanisms inferred from BEVs will be essential for developing next-generation POD prevention strategies.

## Introduction

Delirium is a neurobehavioral syndrome characterized by an alteration of cognitive level; hyper-vigilance, agitation, aggression, rapid change of mood, hallucination and delusion in the hyperactive, whereas lethargy, apathy and confusion in the hypoactive subtype, a common condition among hospitalized older adults especially who undergo surgical procedures[1]. As geriatric population expands, aging-associated disorders including spinal conditions are expected to be commonplace, along with postoperative delirium (POD). Considering the incidence of the POD in type of surgery, 14.5–40.5% of older patients who underwent spinal surgery have developed delirium,^[2–5]^ comparable to the POD after cardiac surgery that led to the most incidence ranged from 28–52%.^[6–8]^ The POD in older patients was shown to be in proportion to mortality, postoperative complications, unplanned intensive care unit admissions, length of hospital stay and non-home discharge[9]. In addition, the adverse outcomes of POD have led to significant economic implication such as high health care expense^[2,10,11]^. Thus, attempts have been made to relieve the difficulties of the patients’ management, and consequently, a prediction model for the POD before surgery has attracted attention[12]. However, the preoperative risk factors significantly associated with the POD status has been inconsistently reported in terms of sex^[13,14]^, proinflammatory markers[15], preoperative cumulative indicators^[16–18]^ and chronic therapy[19]. The discrepancy in the literature raises the possibility of heterogenous study cohorts, resulting in compromising the development of prediction models.

Since the etiologies of the delirium proposed up to date are the perturbation of neurological activities[20], certain bioactive molecules that disturb a cranial nerve system are expected to be the potential candidates for the prediction model. Bacterial extracellular vesicles (BEVs) are such molecules with spheric bilayer lipid membrane derived from bacteria with diameters ranging from 20 to 400 nm, which carry a range of cargo, *e.g.*, lipopolysaccharide, peptidoglycan, nucleic acids and proteins[21]. Recently, the BEVs have been recognized as mediators of crosstalk between gut and brain in the context of cognitive impairment, evidenced by passage through blood-brain barrier (BBB)[22] and inflammation in hippocampus[23]. Since they were shown to be participated in various neurological dysregulation[24], we believe that the BEVs are the promising prognostic as well as functional biomarkers to differentiate clinical outcomes of the POD. However, the prediction of the POD status using preoperative BEVs has not been largely explored.

Under the hypothesis that BEVs from preoperative samples are the prognostic factors of POD in elderly patient cohort, bacterial taxa from circulating BEVs were identified from a unique cohort where baseline characteristics were comparable between patients with and without delirium. We suggest that the profile of preoperative circulating BEVs can contribute to the decision-making to manage patients efficiently in clinical settings, and metabolic cargoes from the BEVs will serve as a resource for the development of therapeutic intervention against the POD.

## Material and methods

### Transparency in the reporting of artificial intelligence

In accordance with the transparency in the reporting of artificial intelligence (TITAN) guideline[25], we declare that no AI tools were employed in any aspect of this study.

### Study cohort

**Figure 1.**
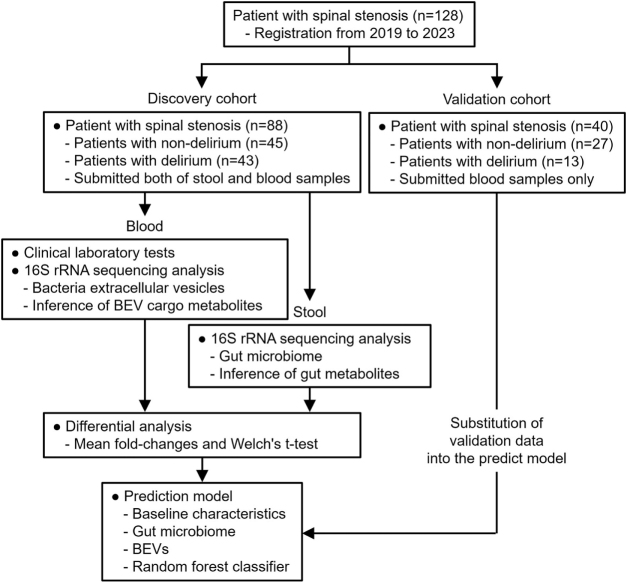
Study flowchart. A total of 128 patients registered for spinal surgery between 2019 and 2023 were grouped into either discovery or validation cohort. Samples from discovery cohort were subjected to clinical laboratory tests and 16S rRNA sequencing analysis to identify and quantify bacterial taxa, and inference of functional pathways. Statistically significant data were used to build a prediction model using a random forest classifier. Validation cohort is an independent data set, not involved in creating the prediction model, for the purpose of ensuring accuracy of the prediction.

This prospective cohort study was conducted at a single tertiary academic hospital in Seoul, South Korea. Our study population (n = 128 with delirium occurrence of 43.8%) (Supplemental Digital Content Table 1, available at: http://links.lww.com/JS9/F609) consisted of a subset of the parent population (n = 536 with delirium occurrence of 17.7%; the details of the population can be found in the materials and methods section of the Supplemental Digital Content)[26] aged ≥70 years who were scheduled to undergo spine surgery between October 2019 and May 2023. Based on sample availability, the 128 patients were divided into discovery and validation cohort; both blood and stool samples were collected from the discovery cohort, whereas only blood samples from the validation cohort (Fig. 1). Patients presenting any of the following conditions were not considered for inclusion: cognitive impairment, as determined by the Mini-Mental State Examination for Dementia Screening (MMSE-DS); diagnosed with a malignant tumor within the last five years; scheduled to undergo surgery with an estimated operation time of less than two hours; history of neurological disorders; or a diagnosis of alcoholism or drug addiction. The primary outcome was postoperative delirium (POD), monitored twice daily from the first to the third postoperative day and once daily from the fourth to the seventh day. If the patients exhibited signs of delirium based on the results of either the Confusion Assessment Method (CAM) or Nursing Delirium Screening Scale (Nu-DESC), experienced physicians further examined them and classified them into the delirium group. All procedures conformed to the standards in the Declaration of Helsinki. Written informed consent was obtained for the study. This study was performed in line with the STROCSS 2025 (STROCSS: Strengthening the Reporting of Cohort, Cross-Sectional, and Case-Control Studies in Surgery) Guidelines[27].

**HIGHLIGHTS**

- The preoperative risk factors involved in postoperative delirium (POD) have been discordantly reported possibly due to heterogeneity of patients, resulting in compromising the development of prediction models.
- The profile of circulating preoperative BEVs is the key prognostic factor for distinguishing POD status in elderly surgical patients with comparable baseline conditions; especially, EVs from Moraxellaceae and Acinetobacter are the most significant prognostic markers for the POD.
- Upon *in silico* analysis, succinate and enterobacterial common antigens delivered from BEVs were expected to participate in pathogenic events, whereas S-methyl-5ʹ-thioadenosine, 2-oxoglutarate, pyruvate, acetate and butyrate may play a neuroprotective role in the POD.
- We suggest that circulating preoperative BEVs is the significant prognostic factors of the POD in elderly patient cohorts, and the defensive and offensive molecular mechanisms inferred in each clinical outcome will be fundamental to develop preventive intervention against the POD.

### Preoperative assessment

Patients’ cognitive function, depression, daily living activities, frailty, nutritional status, and comorbidities were assessed preoperatively using validated geriatric assessment tools. Cognitive function was evaluated with the Korean MMSE-DS, while depression was measured using the Geriatric Depression Scale-Short Form (GDSSF-K). MMSE-DS cut-off scores in this study were adjusted based on education levels, and patients diagnosed with a preoperative MMSE score <23 were excluded from downstream analyses. Functional status was determined by the Korean Activities of Daily Living (K-ADL) and Korean Instrumental Activities of Daily Living (K-IADL) scales, and frailty was assessed using the FRAIL scale. Nutritional status was measured with the Mini Nutritional Assessment-Short Form (MNA-SF), and comorbidities were scored with the Charlson Comorbidity Index (CCI).

### Anesthetic management

All surgical procedures were performed in the prone position. The Wilson frame was used with the head and neck in the neutral position. The types of surgery patients underwent were laminectomy, discectomy, and spinal fusion. Anesthesia was induced with propofol (1–1.5 mg kg^−1^), remifentanil (0.05–0.2 μg kg^−1^ min^−1^), and rocuronium (0.6 mg kg^−1^). Anesthesia was maintained by inhalation or intravenous anesthesia. During surgery, the concentration of sevoflurane, desflurane, or propofol was adjusted to achieve a SedLine® patient state index (PSI) of 25–50, the suggested range for ensuring safety and efficacy of guiding anesthetic administration in general surgical patients by the manufacturer. Vasoactive drugs such as norepinephrine and ephedrine were administered to maintain mean blood pressure within 80–120% of the baseline during the surgery. The lungs were ventilated with a 50% oxygen/air mixture.

### Analysis of circulating BEVs, gut microbiome and inference of metabolic pathways

Steps for the analysis of circulating BEVs, gut microbiome, and inference of metabolites include collection of specimens, DNA extraction, amplification and identification of target DNA sequences, assignment and quantification of bacterial taxonomy, and prediction of BEV cargo metabolites using PICRUSt2. The details can be found in the materials and methods section of the Supplemental Digital Content.

### Statistical analysis

Power analysis to estimate the minimum sample size required for binomial random forest classification were performed with Cohen’s h, a predefined statistical power of 80%, a significant level of 0.05 and locally estimated scatterplot smoothing (Supplemental Digital Content Figure 1, available at: http://links.lww.com/JS9/F609). Significant features were screened and identified by selecting taxa detected in at least 20% of patients, followed by statistical evaluation using Welch’s t-test between two groups. All statistical analyses were performed with 5% significance level, and their consequent visualization was conducted using R version 4.4.2 (R Core Team, R Foundation for Statistical Computing, Vienna, Austria). The details can be found in the materials and methods section of the Supplemental Digital Content.

## Results

### Baseline characteristics of patients with and without delirium

The discovery cohort contained 88 patients (Fig. 1) who underwent spinal surgery; 45 patients did not develop delirium, whereas delirium occurred in 43 patients (48.9%) 67.5 hours on average (median = 48, minimum = 24 and maximum = 216 hours) after spinal surgery (Table 1). Baseline characteristics have been often addressed as preoperative risk factors, strongly associated with the POD[28]. In our cohort, none of the characteristics showed significant differences between patients with and without delirium, except for the number of white blood cells (WBC) and Mean Corpuscular Hemoglobin Concentration (MCHC), significantly more increased in patients with delirium than non-delirium (Table 1). However, considering the normal range of WBC (4.5–11.0 × 10^3^/μL) and MCHC (32–36 g/dL), the statistical significances may not explain the clinical differentiation between the groups. Altogether, the two groups had similar demographic, anthropometric, cognitive function score and clinical laboratory test data. This cohort represents a unique group in which previously considered prognostic factors were well-controlled, making it difficult to identify significant factors using conventional approaches. Therefore, exploring other factors significantly associated with POD status within this cohort is proposed as a downstream analytical strategy.

**Table 1:** Baseline characteristics of discovery cohort

Characteristic	Non-delirium (n = 45)	Delirium (n = 43)	*p-*value
Sex			0.710
Male	12 (13.6%)	14 (15.9%)	
Female	33 (37.5%)	29 (33.0%)	
Age [years]	75.3 ± 3.9	75.6 ± 3.8	0.702
Height [cm]	157.6 ± 7.9	157.3 ± 7.8	0.866
Weight [kg]	60.6 ± 9.4	60.3 ± 9.8	0.911
Body mass [kg/m^2^]	24.3 ± 2.7	24.4 ± 3.6	0.886
Surgical experience			0.999
No	5 (5.7%)	5 (5.7%)	
Yes	40 (45.4%)	38 (43.2%)	
Benzodiazepine Tx			0.101
No	44 (50.0%)	37 (42.1%)	
Yes	1 (1.1%)	6 (6.8%)	
ASA-PS			0.479
I	0 (0.0%)	0 (0.0%)	
II	18 (20.5%)	14 (15.9%)	
III	27 (30.7%)	28 (31.8%)	
IV	0 (0.0 %)	1 (1.1%)	
CCI			0.968
≥4	1 (1.1%)	2 (2.3%)	
<4	44 (50.0%)	41 (46.6%)	
MMSE	27.7 ± 1.9	27.5 ± 1.6	0.593
MoCA	24.1 ± 2.5	23.7 ± 2.8	0.473
GDS	3.6 ± 3.8	4.1 ± 4.2	0.515
WBC [10^3^/μl]	6.2 ± 1.2	6.8 ± 1.7	*0.047*
Hemoglobin [g/dL]	13.1 ± 1.4	13.1 ± 1.5	0.773
Platelet count [10^3^/μl]	241.6 ± 49.2	229.9 ± 49.2	0.268
MCV[fL]	92.1 ± 6.3	92.8 ± 4.2	0.505
MCH [pg]	30.5 ± 2.6	31.2 ± 1.5	0.121
MCHC [g/dL]	33.1 ± 1.1	33.6 ± 0.9	*0.017*
NLR	2.0 ± 1.5	2.2 ± 1.2	0.623
LMR	5.4 ± 2.5	5.2 ± 1.8	0.595
PLR	136.3 ± 71.5	124.8 ± 49.9	0.381
ESR [ml/min/1.73 m^2^]	15.2 ± 15.0	14.0 ± 16.0	0.738
CRP [mg/L]	2.5 ± 8.3	2.9 ± 5.6	0.773
BUN [mg/dL]	19.4 ± 5.5	22.4 ± 12.5	0.159
Creatine [mg/dL]	0.8 ± 0.2	0.9 ± 0.4	0.160
eGFR [ml/min/1.73 m^2^]	74.5 ± 15.2	70.7 ± 18.9	0.305
Total protein [g/dL]	6.9 ± 0.6	7.0 ± 0.4	0.293
Albumin [g/dL]	4.5 ± 0.3	4.4 ± 0.3	0.100

Data are presented with mean ± standard deviation or the number of patients (percentage).

ASA-PS, the American Society of Anesthesiologists-physical status classification system; BUN, Blood Urea Nitrogen; CCI, Charlson Comorbidity Index; CRP, C-Reactive Protein; ESR, Erythrocyte Sedimentation Rate; eGFR, Estimated Glomerular Filtration Rate; GDS, Geriatric Depression Scale; LMR, Lymphocyte to Monocyte Ratio; MMSE, Mini-Mental State Examination; MoCA, Montreal Cognitive Assessment; MCV, Mean Corpuscular Volume; MCHC, Mean Corpuscular Hemoglobin Concentration; MCH, Mean Corpuscular Hemoglobin; NLR, Neutrophil to Lymphocyte Ratio; PLR, Platelet to Lymphocyte Ratio.

### Profiles of bacterial extracellular vesicles between the POD status

**Figure 2.**
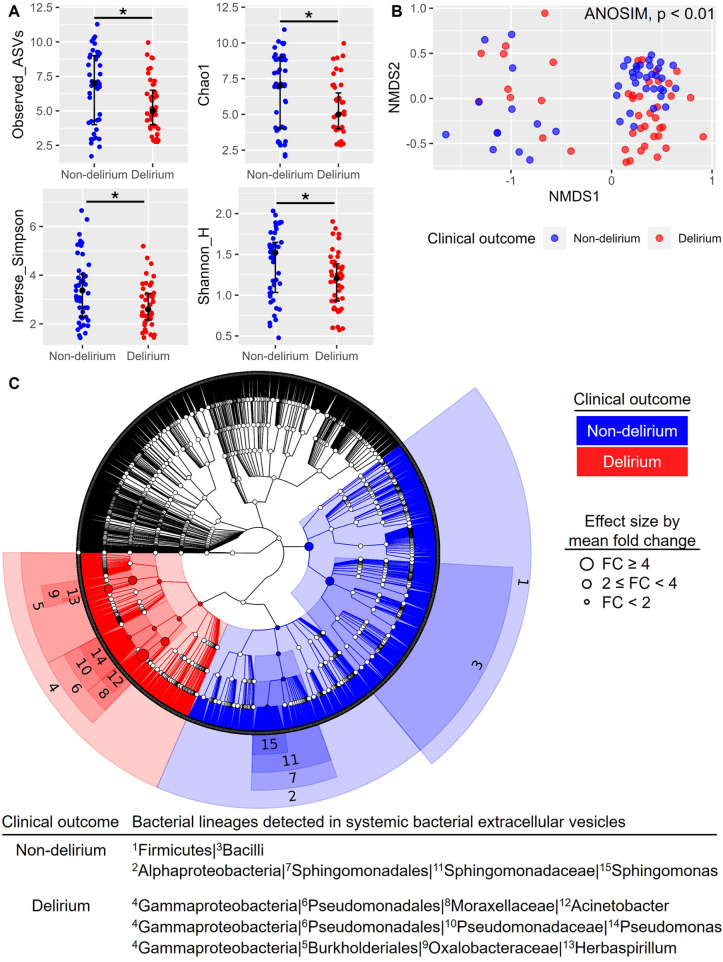
Diversity and bacterial taxa of circulating BEVs between the POD status. (A) The α-diversity was measured by the observed number of amplicon sequence variants (ASVs) and Chao1 index for richness, and by inverse Simpson and Shannon H for evenness. Statistical significance between non-delirium (blue, n = 45) and delirium (red, n = 43) was tested by Welch’s t-test. Data were expressed with individual raw data, median ± interquartile range and level of significance: *, *P* < 0.05. (B) Non-metric dimensional scaling (NMDS) ordinations based upon Bray-Curtis dissimilarity using all ASVs were used to understand β-diversity. Statistical significance between non-delirium (blue, n = 45) and delirium (red, n = 43) was estimated by analysis of similarity (ANOSIM), resulting in *P* < 0.01. (C) Significant bacterial taxa were organized on a cladogram based on mean fold changes (FC, classified as follows: greater than or equal to 4; between 2 and 4 (inclusive of 2 but exclusive of 4); and less than 2) to associate clinical outcomes (i.e., blue for non-delirium and red for delirium) with bacterial lineages.

Bacterial extracellular vesicles (BEVs) have been considered to deliver messages from gut environment to extraintestinal organs including brain^[29,30]^. To understand the BEVs as potential prognostic markers of the POD, we analyzed the sequences of 16S rRNA genes of the BEV isolated from blood sampled before surgery. For the diversity of BEVs within a group (Fig. 2A), patients with delirium had lower levels of richness (measured by observed amplicon sequence variants (ASVs) and Chao1) and evenness (measured by Shannon H and Inverse Simpson) compared to patients without delirium, indicating a lower number and diversity of BEVs in preoperative blood from patients with delirium. A nonmetric multidimensional scaling ordination using all ASVs was used to understand the diversity of BEVs between the groups, resulting in significantly different systemic BEV composition between patients with and without delirium (Fig. 2B). To link the BEVs with the POD status, significantly different bacterial taxa from the blood samples (Supplemental Digital Content Figure 2, available at: http://links.lww.com/JS9/F609) were visualized with cladogram (Fig. 2C). At the class level, the BEVs from Bacilli and Alphaproteobacteria were more abundant in non-delirium, whereas the BEVs from Gammaproteobacteria were more detected in delirium group (Fig. 2C). Especially, Acinetobacter genus of Gammaproteobacteria lineage most significantly differentiated the clinical outcomes; the relative abundance in delirium was more than 4 times higher than that in non-delirium group (Fig. 2C). These data revealed that the profile of BEVs in preoperative blood is significantly associated with the POD status.

### Association of bacterial extracellular vesicles and gut microbiome

To understand the BEVs as derivatives of gut microbes, the sequences of 16s rRNA gene obtained from stools were analyzed under the hypothesis that the profile of significant gut microbial taxa is comparable to that of the systemic BEVs shown in Figure 2. However, the analysis of microbial diversity failed to reach statistical significance (Supplemental Digital Content Figure 3A and B, available at: http://links.lww.com/JS9/F609). In addition, few gut bacterial taxa were associated with clinical outcomes; merely two taxa, Peptococcales order and Peptococcaceae family were more detected in patients with delirium, and there was no taxon more abundant in patients with non-delirium (Supplemental Digital Content Figure 3C and D, available at: http://links.lww.com/JS9/F609). The strength and direction of association between significant BEVs and gut microbes were measured using Pearson correlation, resulting in no strong correlation; if any, coefficients were only 0.22 and 0.26 (Supplemental Digital Content Figure 4, available at: http://links.lww.com/JS9/F609). Thus, the profile of systemic BEVs appears to be independent of gut microbial communities in our study cohort.

### Random forest classifier with significant BEVs most accurately predicts POD status

**Figure 3.**
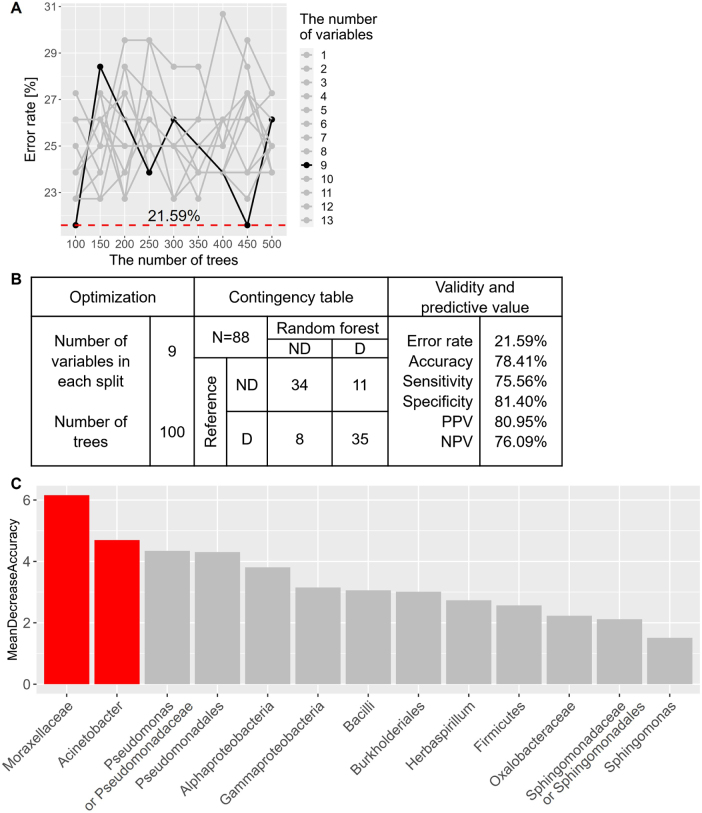
Optimization of random forest classifier and its variable importance. (A) Error rates were visualized according to the number of trees and variables. Random forest classifier with 9 variables and 100 trees (black solid line) were selected for prediction model at the level of 21.59% error rate (red dashed line). (B) Validity and predictive values were calculated based upon the contingency table of random forest classifier with 9 variables and 100 trees. (C) Variable importance was arranged in descending order of mean decrease in accuracy from the optimized random forest prediction model. Mean decrease accuracy is the measure of the performance of the model without each bacterial taxonomy; removal of high ranked taxonomy causes the model to lose accuracy. The two variables that are of the highest importance were red-highlighted.

To establish prediction model for the POD status, random forest classifiers were constructed with the significant factors of clinical laboratory tests, gut microbes or BEVs. Compared to that with the factors of clinical laboratory tests or gut microbiome (Supplemental Digital Content Figure 5A and B, available at: http://links.lww.com/JS9/F609), the random forest classifier with the significant BEVs showed the lowest prediction error rate, 21.59%, measured by Out-of-bag (OOB) error with 100 trees and 9 variables (Fig. 3A and B). Of the15 significant BEVs, Moraxellaceae and Acinetobactor were the top 2 important taxa across the 100-decision trees (Fig. 3C).

**Figure 4.**
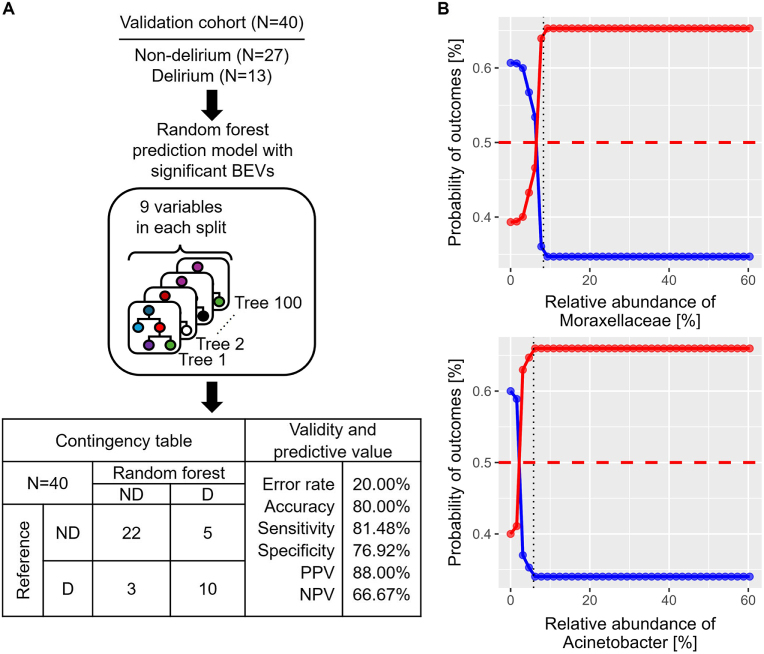
Validation of the prediction model with independent data. (A) Clinical outcomes of validation cohort, composed of 27 non-delirium and 13 delirium patients were predicted by the optimized random forest classifier. Validity and predictive value of random forest prediction model in validation set were summarized in contingency table. (B) Partial dependence plots were constructed to understand the relationship between the relative abundance of the two most important taxa from BEVs, i.e., Moraxellaceae and Acinetobacter, and probability of each clinical outcome, non-delirium (blue) and delirium (red). Black dotted lines indicate the minimum relative abundance at which the maximum probabilities of delirium are reached for each taxon. Additionally, the red dashed lines indicate 50% probability of outcomes.

To understand the practicability of the prediction model in clinical setting, the random forest classifier was validated with external data, composed of 40 patients; 13 patients showed delirium, and 27 patients did not after surgery (Fig. 4A; Supplemental Digital Content Table 2, available at: http://links.lww.com/JS9/F609). The prediction model correctly classified 32, but misclassified 8 out of 40 patients, resulting in 80.00% accuracy (Fig. 4A; Supplemental Digital Content Table 3, available at: http://links.lww.com/JS9/F609). In addition, partial dependence plots were constructed to analyze how the relative abundance of each BEV impacts the prediction of random forest (Fig. 4B; Supplemental Digital Content Figure 6, available at: http://links.lww.com/JS9/F609). Of the significant factors, two taxa from BEVs showed the highest probabilities of prediction of the POD status despite their low relative abundance; patients with ≥5.8% Acinetobacter and ≥8.3% Moraxellaceae EVs (black dotted line) in preoperative blood samples were more likely to be delirious after surgery with a probability of 66 and 65%, respectively (Fig. 4B). These data suggest that preoperative circulating EVs from Moraxellaceae or Acinetobacter are the most significant prognostic marker for the POD.

### The inference of BEV cargo metabolites that potentially regulate the POD status

**Figure 5.**
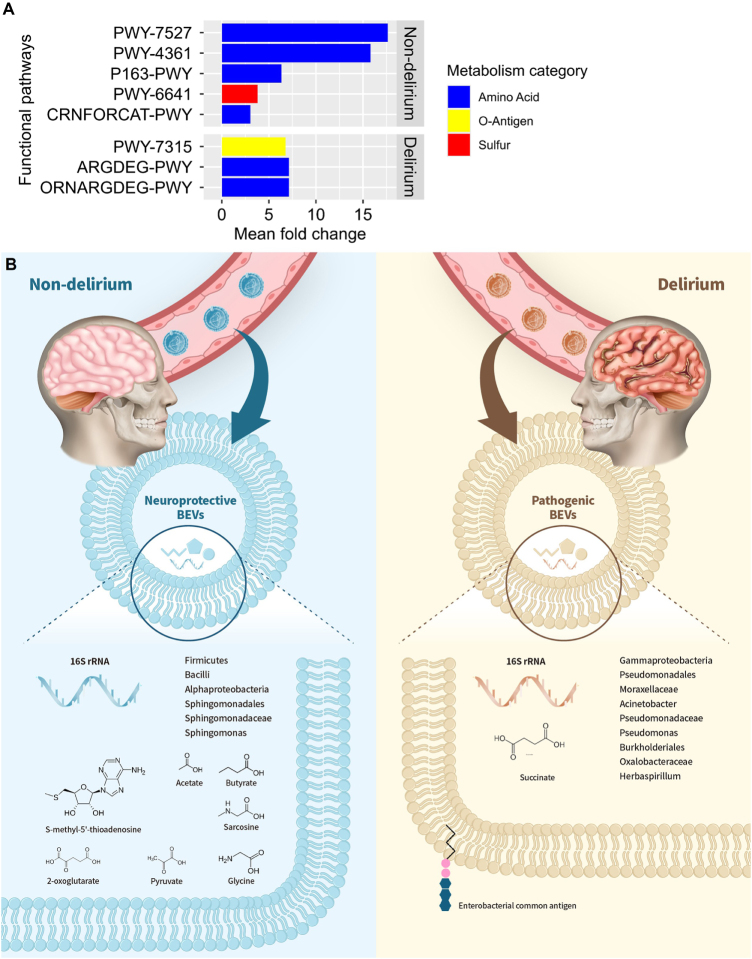
The inference of significant functional pathways and cargo metabolites from BEVs. (A) Functional pathways based upon 16S rRNA gene sequencing of circulating BEVs that reached statistical significance with mean fold change >3 between clinical outcomes were considered the significant functional pathways. Eight significant functional pathways were identified; 5 functional pathways were expected to be more abundant in patients with non-delirium, whereas patients with delirium appeared to be enriched with 3 functional pathways. (B) Schematic presentation of bacterial taxa and predicted cargo metabolites potentially involved in neuroprotective or pathogenic mechanisms of the POD.

To understand the potential mechanism of the effect of BEVs on the POD status, the cargo metabolites that BEVs can deliver were inferred based upon the analysis of 16s rRNA gene sequencing from blood samples, and relative abundance of the functional genes were aggregated into metabolic pathways[31]. Patients with non-delirium and delirium were expected to be associated with five and three functional pathways, respectively (Fig. 5A; Table 2; Supplemental Digital Content Figure 7, available at: http://links.lww.com/JS9/F609). Considering the metabolites produced from the pathways, nine metabolites were expected to regulate the clinical outcomes; S-methyl-5ʹ-thioadenosine (MTA)^[32,33]^ from PWY-7527, 2-oxoglutarate[34] from PWY-4361, acetate and butyrate^[35,36]^ from P163-PWY, pyruvate[37] from PWY-6641, and sarcosine[38] and glycine[39] from CRNFORCAT-PWY appeared to be neuroprotective, whereas accumulation of succinate[40] from ORNARGDEG-PWY and ARGDEG-PWY, and enterobacterial common antigen[41] from PWY-7315 may participate in pathogenic mechanisms of the POD (Fig. 5B). The profile of metabolites inferred here can be considered the research resource to investigate defensive and offensive molecular mechanisms in each clinical outcome.

**Table 2:** Functional pathways inferred by PICRUSt2

Functional pathways	Description
PWY-7527	L-methionine salvage cycle III
PWY-4361	S-methyl-5-thio-α-D-ribose 1-phosphate degradation
P163-PWY	L-lysine fermentation to acetate and butanoate
PWY-6641	Superpathway of sulfolactate degradation
CRNFORCAT-PWY	Creatinine degradation I
PWY-7315	dTDP-N-acetylthomosamine biosynthesis
ARGDEG-PWY	Superpathway of L-arginine, putrescine, and 4-aminobutanoate degradation
ORNARGDEG-PWY	Superpathway of L-arginine and L-ornithine degradation

## Discussion

Preoperative risk factors have been investigated to forecast the delirium after spinal surgery; female patients, history of surgery[2], benzodiazepine use, lower hemoglobin concentration[42], lower Mini-Mental State Examination score[43], higher ASA score[4] and higher C-reactive protein (CRP)[5] were strongly associated with higher chance of delirium. However, none of the risk factors differentiated the POD status in our study cohort. This discrepancy raises the possibility of heterogenous patient population with same phenotype, and conventional approaches may not correctly predict the POD status. Our group has paid attention to BEVs as a resource of the novel prognostic markers under the rationale that BEVs embedded with pathogen-associated molecular patterns activate innate immunity, leading to inflammatory cytokines[44], BEVs cross blood-brain barrier[45], and the alteration of BEV profiles is in parallel with dysbiosis of gut microbiome in mouse model of neurodegenerative diseases[46]. This novel approach effectively conveyed key points: preoperative circulating BEVs well-classified the POD status where baseline characteristics were comparable.

Acinetobacter BEVs were demonstrated one of the important features and a significant impact on prediction of the POD, reflecting effector function of bioactive molecules in the BEVs that may render patients susceptible to the POD. The possible pathogenic mechanisms of the BEVs may be associated with outer membrane protein A (OmpA) embedded in Acinetobacter baumannii vesicles; the OmpA induced mitochondrial fragmentation and elevated reactive oxygen species, resulting in cytotoxicity *in vitro*[47]. The mitochondrial fragmentation were shown to be significantly associated with neurodegenerative diseases including delirium[48]; the excessive mitochondrial fragmentation and disturbed mitochondrial morphogenesis were detected in the hippocampus and prefrontal cortex of aged mice that underwent surgery and anesthesia, along with delirium-like behavior[49]. In addition, Acinetobacter species have been implicated in bloodstream infection in clinical settings[50]. Thus, the circulating Acinetobacter BEVs may result from blood-borne dissemination, rather than translocation from gut lumen to circulatory system, a possible reason for different profiles between bacterial taxa in BEVs and gut environment in our study cohort.

Given the varied cargoes carried by BEVs as regulators of the neurological functions[51], MTA, 2-oxoglutarate, shot chain fatty acids, pyruvate, sarcosine and glycine may be neuroprotective druggable metabolites according to previous publication. It was demonstrated experimentally that preoperative treatment of MTA prevented inflammation and cognitive decline induced by anesthesia and surgery^[32,33]^, and low levels of systemic MTA were significantly associated with postoperative delayed neurocognitive recovery in patients who underwent neck and maxillofacial tumor resection[33]. Treatment with 2-oxoglutarate effectively prevented seizure-like behaviors in mice with severe brain mitochondrial DNA damage induced by kainic acid[34]. The pretreatment of SCFAs including acetate and butyrate significantly improved impaired cognitive function, along with decreased proinflammatory cytokines in sepsis-associated encephalopathy mice[35]. In addition, butyrate-supplementation significantly reduced the amyloid plaque formation in parallel with an increase of cognitive memory performance in mice model of Alzheimer’s disease[36]. The beneficial effect of pyruvate was demonstrated with varied neuropathogenic models, featured with reduction of neuronal damage, hyperexcitability, pro-inflammatory cytokines and memory deficits[37]. Sarcosine has been shown to be neuroprotective in experimental Alzheimer’s disease model; neurofibrillary tangles in brain were significantly attenuated, along with higher levels of antioxidants and lower expression levels of TNF-α[38]. Lastly, glycine participated in neuroprotection against oxidative stress-mediated neurodegeneration and memory impairment in experimental aging model[39]. The BEV cargo metabolites may play not only a defensive, but also an offensive role in cognitive function. Succinate may precipitate cognitive impairment; neonatal mice under hypoxia environment significantly increased succinate levels in cortex and hippocampus, followed by learning and memory defects[40]. Moreover, the inhibition of succinate accumulation reversed the neuronal injury, and consequently improved cognitive deficits[40]. The dTDP-N-acetylthomosamine, one of the building blocks of enterobacterial common antigen (ECA) was demonstrated as an immunomodulatory antigen; ECA-stimulated lymphocytes from patients with ulcerative colitis markedly inhibited macrophage migration[52]. Microglial cells, specialized macrophages in the central nervous system migrate toward amyloid-beta (Aβ) plaque, and leads to Aβ clearance[53]. Since an increase of postoperative Aβ can accelerate the risk of POD[54], the ECA-mediated inhibition of microglia migration toward Aβ plaque may partially explain the mechanisms of cognitive impairment of the POD patients.

There are limitations to be supplemented and refined in the following investigation. First, data was generated from a single-center study. To rigorously establish the BEVs as universal biomarkers, further investigation should be conducted with multicenter large cohort. Second, subclinical outcomes, *e.g.*, hyperactive, hypoactive and mixed delirium should be included to be more informative for clinical settings. Third, the POD is a known risk factor of postoperative cognitive decline (POCD, characterized by later onset, persistent cognitive decline and subtle cognitive impairment)[55]. Thus, the long-term effect of BEVs on POCD in patients with a history of POD should be investigated longitudinally to improve clinical decision-making. Fourth, since it can significantly differentiate the POD status, different routes of anesthetic techniques (i.e., inhalational or intravenous administration) should be considered to improve the prediction model in future analyses. Fifth, the analysis of BEVs as a clinical approach is still an area of ongoing investigation, because of time-intensive process. Lastly, since reliance on *in silico* analysis of functional pathways is inconclusive, follow-up study should include advanced methodologies to scrutinize the effect of metabolites on the POD pathogenesis.

In conclusion, this investigation is the first to demonstrate that preoperative circulating BEVs serve as prognostic biomarkers for postoperative delirium after spinal surgery in elderly patient cohort where two groups of clinical outcomes have similar baseline characteristics. In addition, we suggest that the random forest classifier with the significant BEVs can contribute to the decision-making to manage patients efficiently in clinical settings, and metabolic cargoes inferred from the BEVs (Fig. 5B) will serve as a resource for the development of therapeutic intervention against the POD.

## Data Availability

All data are available upon reasonable request from the corresponding author.

## Research registration unique identifying number (UIN)

ClinicalTrials.gov Identifier: NCT04120272.
